# Seroprevalence and dynamics of anti-SARS-CoV-2 antibodies: a longitudinal study based on patients with underlying diseases in Wuhan

**DOI:** 10.1186/s12931-022-02096-5

**Published:** 2022-07-15

**Authors:** Jin Yang, Libing Ma, Li Guo, Ting Zhang, Zhiwei Leng, Mengmeng Jia, Fangyuan Chen, Weiran Qi, Xingxing Zhang, Qing Wang, Yuan Yang, Luzhao Feng, Lili Ren, Weizhong Yang, Chen Wang

**Affiliations:** 1 School of Population Medicine and Public Health, Chinese Academy of Medical Sciences and Peking Union Medical College, Beijing, China; 2 Institute of Pharmaceutical and Medical Devices Supervision, National Medical Products Administration-Chinese Academy of Medical Sciences, Beijing, China; 3 Department of Respiratory and Critical Care Medicine, Affiliated Hospital of Guilin Medical University, Guilin, China; 4 National Health Commission Key Laboratory of Systems Biology of Pathogens and Christophe Mérieux Laboratory, Institute of Pathogen Biology, Chinese Academy of Medical Sciences and Peking Union Medical College, Beijing, China; 5 Key Laboratory of Respiratory Disease Pathogenomics, Chinese Academy of Medical Sciences and Peking Union Medical College, Beijing, China

**Keywords:** COVID-19, SARS-CoV-2, Underlying diseases, Antibody, Single natural infection

## Abstract

**Background:**

Assessing the humoral immunity of patients with underlying diseases after being infected with SARS-CoV-2 is essential for adopting effective prevention and control strategies. The purpose of this study is to analyze the seroprevalence of people with underlying diseases and the dynamic change features of anti-SARS-CoV-2 antibodies.

**Methods:**

We selected 100 communities in Wuhan using the probability-proportional-to-size sampling method. From these 100 communities, we randomly selected households according to a list provided by the local government. Individuals who have lived in Wuhan for at least 14 days since December 2019 and were ≥ 40 years old were included. From April 9–13, 2020, community staff invited all selected individuals to the community healthcare center in batches by going door-to-door or telephone. All participants completed a standardized electronic questionnaire simultaneously. Finally, 5 ml of venous blood was collected from all participants. Blood samples were tested for the presence of pan-immunoglobulins, IgM, IgA, and IgG antibodies against SARS-CoV-2 nucleocapsid protein and neutralising antibodies were assessed. During the period June 11–13, 2020 and October 9–December 5, 2020, all family members of a positive family and matched negative families were followed up twice.

**Results:**

The seroprevalence of anti-SARS-CoV-2 antibodies in people with underlying diseases was 6.30% (95% CI [5.09–7.52]), and that of people without underlying diseases was 6.12% (95% CI [5.33–6.91]). A total of 313 people were positive for total antibodies at baseline, of which 97 had underlying disease. At the first follow-up, a total of 212 people were positive for total antibodies, of which 66 had underlying disease. At the second follow-up, a total of 238 people were positive for total antibodies, of which 68 had underlying disease. A total of 219 participants had three consecutive serum samples with positive total antibodies at baseline. The IgG titers decreased significantly with or without underlying diseases (*P* < 0.05) within the 9 months at least, while the neutralizing antibody titer remained stable. The titer of asymptomatic patients was lower than that of symptomatic patients (baseline, *P* = 0.032, second follow-up, *P* = 0.018) in the underlying diseases group.

**Conclusion:**

Our research focused on the serological changes of people with and without underlying diseases in a state of single natural infection. Regardless of the underlying diseases, the IgG titer decreased significantly over time, while there was no significant difference in the decline rate of IgG between with and without underlying diseases. Moreover, the neutralizing antibody titer remained relatively stable within the 9 months at least.

**Supplementary Information:**

The online version contains supplementary material available at 10.1186/s12931-022-02096-5.

## Background

The global COVID-19 pandemic that started in 2019 is the most extensive to afflict humanity in a century. It is a serious health crisis and challenge worldwide. Some studies have shown that the risk of severe COVID-19 is related to underlying diseases, such as hypertension, diabetes, and cardiovascular and cerebrovascular diseases [1, 2]. A study published in BMJ that analyzed 113 deaths and 161 patients diagnosed with COVID-19 showed that 63% of the deceased and 39% of the recovered patients had at least one underlying chronic disease [3]. The results of another study published by the Lancet, in which severe COVID-19 patients in two hospitals in the United States were analyzed, showed that more than 80% of the patients had at least one chronic disease, of which hypertension and diabetes were most common [4].

Considering the spread of COVID-19 in countries around the world, the mutation of virus strains, and the development and application of vaccines, assessing the proportion of the infected and immunized population with and without underlying diseases is essential to determine effective prevention and control strategies with the ultimate goal of preventing the epidemic from expanding continuously [5–7].

In a previous study, we analyzed the dynamic changes of seroprevalence and humoral immune response after natural infection with COVID-19 in Wuhan [6]. However, an in-depth study on patients with underlying diseases is lacking. The purpose of this study is to investigate the seroprevalence of people with underlying disease and the characteristics of dynamic changes in anti-SARS-CoV-2 antibodies.

## Methods

### Study design and participants

This was a population-level and longitudinal study. In this study, a multistage, population-stratified, and cluster random sampling method was adopted to recruit participants from Wuhan, which is comprised of 13 districts. The probability-proportional-to-size (PPS) method was used in the sampling design $\left({\mathrm{W}}_{\mathrm{base}}=\frac{\mathrm{Number of residents in the district}}{\mathrm{Total population in sampled communities}\times \mathrm{number of communities}}\right)$.

First, we selected 100 communities using the PPS sampling method. Then, we randomly selected households from the 100 communities according to the list of households provided by the local government. Individuals who have lived in Wuhan for at least 14 days since December 2019 and were ≥ 40 years old were included. Individuals who could not be obtained or refused to participate were excluded.

From April 9 to 13, 2020, community staff invited all selected individuals to the community healthcare center for investigation in batches by going door-to-door or telephone. The participants signed a written informed consent form after fully understanding the content and significance of the survey. If the participant was illiterate or unable to sign, it could be signed by someone else with the participant’s consent. All participants completed a standardized electronic questionnaire simultaneously. Finally, 5 ml of venous blood was collected from all participants. A family with one or more individuals who were positive for antibodies against SARS-CoV-2 at baseline was defined as a positive family. A negative family was defined as a household of which all of the family members tested negative for SARS-CoV-2 and lived next-door to a positive family. For each included positive family, two location-matched negative families were included at baseline.

During the period June 11–13, 2020, and October 9–December 5, 2020, all family members of a positive family and matched negative families were followed up twice. Similarly, the trained investigators also asked and collected the information from the participants.

We finally divided the participants into an underlying disease group and no underlying disease group. Participants with at least one underlying disease, such as lung diseases (asthma, COPD, pulmonary heart disease, pulmonary fibrosis, etc.), hypertension, diabetes, cardiovascular and cerebrovascular diseases, chronic kidney disease, chronic liver disease, tumor, or immunodeficiency diseases were classified as underlying disease group. The trained volunteer confirmed with the participants whether the underlying conditions have been diagnosed by doctors.

### Variables

Basic information included area, location of investigation, current address, gender, date of birth, ID number, occupation, and whether the participant was a smoker or non-smoker.

Basic medical history included whether the participants have underlying conditions. When recording this information, the trained investigator confirmed with the participants whether the underlying conditions have been diagnosed by doctors.

To trace the contacts and exposure, onset, and outcome during the COVID-19 epidemic the following questions were included: “Have you had the following clinical symptoms since December 2019?”; “Have you visited a medical institution because of fever or respiratory diseases since December 2019?”; “Have you been diagnosed with COVID-19 since December 2019 and if so, when?”; “Have you had the following travel or residence history since December 2019 and if so, when?”; “Have you been in contact with anyone with fever or respiratory symptoms since December 2019 and if so, when?”; “Have you ever been exposed to confirmed cases of COVID-19 since December 2019 and if so, when?”; “Have you ever been exposed to asymptomatic SARS-CoV-2 infections since December 2019 and if so, when?”; and “Is the respondent a COVID-19 confirmed case who is recorded in the Chinese Notifiable Infectious Diseases Information System?” (Additional file 2).

Confirmed COVID-19 cases were determined through self-report in the baseline questionnaire of a diagnosis according to the Chinese Clinical Guidance, with quantitative PCR assay positive for SARS-CoV-2 and lung CT scan. Symptomatic infection was defined as self-reported fever or respiratory symptoms and positive SARS-CoV-2 antibodies. Positive SARS-CoV-2 antibodies since December 1, 2019, and no self-reported relevant symptoms were defined as asymptomatic infection.

### Laboratory measurements

Serum samples were separated in the Wuhan Center for Disease Control & Prevention laboratory within 8 h after collecting the blood samples. All laboratory tests on the blood samples were performed at the Christophe Mérieux Laboratory, Institute of Pathogen Biology, Chinese Academy of Medical Sciences & Peking Union Medical College in Beijing, China. All serum samples were inactivated at 56 °C for 30 min before use, and recombinant N protein was used as the detection antigen. The electrochemiluminescence immunoassay (ECLIA, Roche Diagnostics, Rotkreuz, Switzerland) kit was used to detect total antibodies before antibody typing. The ELISA method was used to detect titers of IgA, IgM, and IgG antibodies against the SARS-CoV-2-N protein in serum. The multifunctional microplate reader SpectraMax M5 (Molecular Devices, Sunnyvale, CA, USA) was used to measure the optical density at 450 nm (OD_450_). The cutoff value for IgG, IgA, and IgM were determined to be 0.10, 0.20, and 0.30, respectively, by calculating the average OD_450_ value of negative serum samples plus 3 times the standard deviation value. The ECLIA and ELISA assays were further validated with other serum samples. In-house microneutralization assays were used to detect neutralizing antibodies. The Reed-Muench method was used to calculate the neutralizing antibody titers, of which 1:8 was defined as the cutoff.

### Statistical analysis

Continuous variables were tested for normality. The two groups of continuous variables that were not normally distributed were compared using a nonparametric test (two independent samples). The Kruskal–Wallis nonparametric test was used for three groups of continuous variables with non-normal distribution. Categorical variables were expressed as percentages. The χ^2^ test or Fisher’s exact test was applied to compare categorical variables. The binary logistic regression (stepwise method) was used to investigate the influencing factors. Only statistically significant variables in the univariate logistic regression models were further analyzed in multivariate logistic regression models. All statistical tests were performed two-sided using R software and SAS software (version 9.4), and *P* < 0.05 was considered as statistically significant.

### Role of the funding source

The funders of the study had no role in study design, data collection, data analysis, data interpretation, or writing of the report.

## Results

### Baseline demographics and clinical characteristics

The baseline demographics and clinical characteristics of the participants can be found in Table 1. A total of 5,067 people were included in this study, of which 313 people were positive for total antibodies (positive rate 6.18% [95%, CI: 5.51–6.84]). Among them, 767 people were in the age 56–60 group, of which 63 people had positive total antibodies (positive rate 8.21%); and 827 people were in the age ≥ 66 group, of which 63 people had positive total antibodies (positive rate 7.62%). There were 2492 males, of which 124 had positive total antibodies (positive rate 4.98%), and 2575 females, of which 189 had positive total antibodies (positive rate 7.34%). There were 1,476 retirees, of which 138 had positive total antibodies (the highest positive rate of 9.35%) and a total of 38 health workers, of which 3 had positive total antibodies (positive rate 7.89%). There were 4875 people who self-reported no symptoms, of which 248 were asymptomatic (positive rate 5.09%) and 192 people who self-reported that they had symptoms, of which 65 were symptomatic (positive rate 33.85%). There were 3528 people without underlying disease, of which 216 had positive total antibodies (positive rate 6.12%, 95%, CI: 5.33–6.91), and there were 1539 people with underlying disease, of which 97 had positive total antibodies (positive rate 6.30%, 95%, CI: 5.09–7.52).

**Table 1:** Baseline demographics and positive rate of total antibodies of study subjects

Variables	Number of population (%)	Number of positive total antibody (%)	95% CI	*P*-value
Total	5067 (100%)	313 (6.18%)	5.51–6.84	
Age (years)				
40–45	1072 (21.16%)	57 (5.32%)	3.97–6.67	0.02
46–50	924 (18.24%)	48 (5.19%)	3.76–6.63	
51–55	737 (14.55%)	45 (6.11%)	4.37–7.84	
56–60	767 (15.14%)	63 (8.21%)	6.27–1.02	
61–65	740 (14.60%)	37 (5.00%)	3.43–6.57	
≥ 66	827 (16.32%)	63 (7.62%)	5.81–9.43	
Sex				
Male	2492 (49.18%)	124 (4.98%)	4.12–5.83	< 0.001
Female	2575 (50.82%)	189 (7.34%)	6.33–8.35	
Occupation				
Commercial service personal	546 (10.78%)	28 (5.13%)	3.27–6.98	< 0.001
Workmen/farmers	1270 (25.06%)	40 (3.15%)	2.19–4.11	
Others	1312 (25.89%)	87 (6.63%)	5.28–7.98	
Community workers	425 (8.39%)	17 (4.00%)	2.13–5.87	
Retirees	1476 (29.13%)	138 (9.35%)	7.86–1.08	
Health workers	38 (0.75%)	3 (7.89%)	0.00–1.69	
Smoke				
No	3392 (66.94%)	240 (7.08%)	6.21–7.94	< 0.001
Yes	1446 (28.54%)	53 (3.67%)	2.70–4.64	
Ever smoked	229 (4.52%)	20 (8.73%)	5.05–1.24	
Self-reported symptom				
No	4875 (96.21%)	248 (5.09%)	4.47–5.70	< 0.001
Yes	192 (3.79%)	65 (33.85%)	27.10–40.61	
To the hospital for fever or respiratory symptoms since December 2019				
No	4946 (97.61%)	258 (5.22%)	4.60–5.84	< 0.001
Yes	121 (2.39%)	55 (45.45%)	36.45–54.45	
Contact with anyone with fever or respiratory symptoms since December 2019				
No	4821 (95.15%)	262 (5.43%)	4.79–6.07	< 0.001
Yes	246 (4.85%)	51 (20.73%)	15.63–25.83	
Contact with a SARS-CoV-2 confirmed case since December 2019				
No	4922 (97.14%)	280 (5.69%)	5.04–6.34	< 0.001
Yes	145 (2.86%)	33 (22.76%)	15.85–29.66	
Underlying disease				
No	3528 (69.63%)	216 (6.12%)	5.33–6.91	0.81
Yes	1539 (30.37%)	97 (6.30%)	5.09–7.52	

Underlying diseases defined as: at least one disease, such as hypertension, pulmonary disease, cancer (undergoing chemotherapy), diabetes, cardiovascular disease, chronic kidney disease, chronic liver disease, and immunodeficiency disease, among others. Workmen are men employed to do manual labor

### Analysis of risk factors for SARS-CoV-2 infection

Our results showed that underlying disease was not a risk factor for positive SARS-CoV-2 (SARS-CoV-2 patients without underlying disease vs. SARS-CoV-2 patients with underlying disease, OR = 0.90, 95%, CI: 0.71–1.14) (Additional file 2: Table S1). We further conducted a stratified analysis to study the risk factors of SARS-CoV-2 positive patients with underlying diseases. The results show that retirees (vs. other occupations, OR = 2.71, 95% CI: 1.40–5.26), health workers (vs. other occupations, OR = 17.76, 95% CI: 3.32–94.95), people who have been exposed to fever or respiratory symptoms since December 2019 (vs. people who have not been exposed, OR = 5.78, 95% CI: 2.99–11.18) were more likely to be infected with SARS-CoV-2, as shown in Additional file 2: Table S2.

### Dynamic changes of antibodies over time

A total of 313 people were positive for total antibodies at baseline, of which 216 had no underlying disease and 97 had underlying disease. At the first follow-up, a total of 212 people were positive for total antibodies, of which 146 had no underlying disease and 66 had underlying disease. At the second follow-up, a total of 238 people were positive for total antibodies, 170 of whom had no underlying disease and 68 had underlying disease. Regardless of whether underlying disease was present, the positive rate of IgA and IgM declined rapidly over time. In people with no underlying disease, the proportion of positive neutralizing antibodies increased over time. In people with underlying disease, the proportion of positive neutralizing antibodies increased from baseline to the first follow-up, while it decreased slightly during the period from the first follow-up to the second follow-up (Table 2).

**Table 2:** When the total antibody is positive, the change in the number of IgA, IgM, IgG, Nabs positive patients with and without underlying disease

Total antibody positive population (No.)	Underlying disease or not (No.)	IgA + (%)	IgM + (%)	IgG + (%)	Nabs + (%)
Baseline (313)	No (216)	43 (19.91%)	26 (12.04%)	216 (100.00%)	92 (42.59%)
	Yes (97)	22 (22.68%)	7 (7.22%)	97 (100.00%)	43 (44.33%)
First follow-up (212)	No (146)	16 (10.96%)	4 (2.74%)	145 (99.32%)	67 (45.89%)
	Yes (66)	11 (16.67%)	1 (1.52%)	66 (100.00%)	35 (53.03%)
Second follow-up (238)	No (170)	11 (6.47%)	3 (1.76%)	168 (98.82%)	88 (51.76%)
	Yes (68)	6 (8.82%)	1 (1.47%)	63 (92.65%)	36 (52.94%)

*Nabs* neutralising antibody titres

**Fig. 1:**
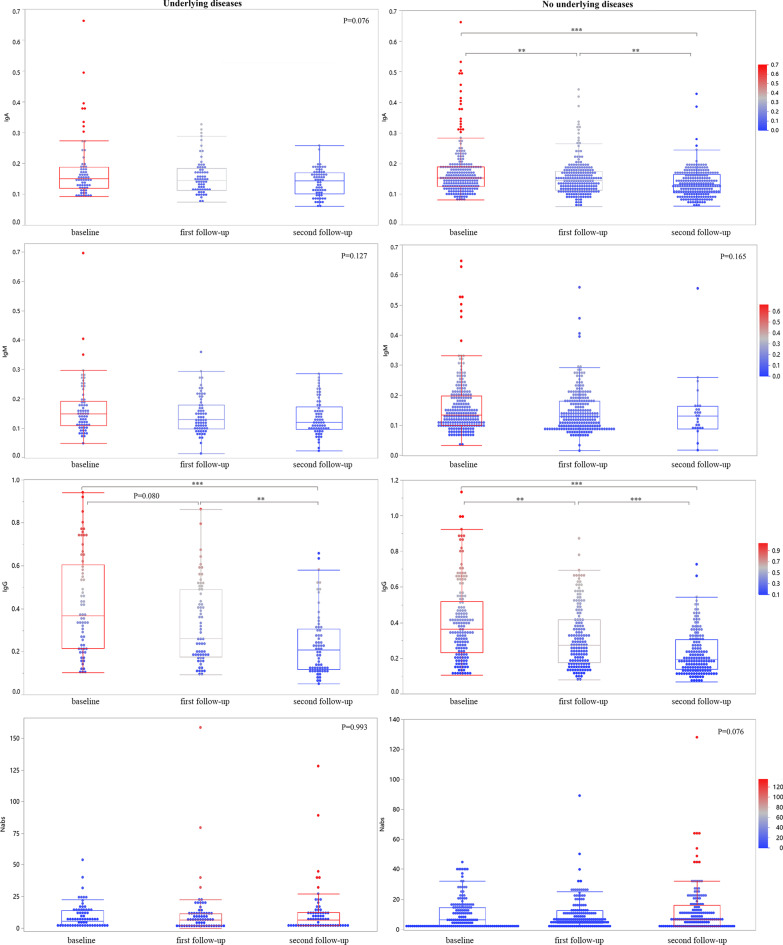
Comparison of antibody titers of patients with and without underlying disease among 219 subjects with positive total antibodies at baseline and three consecutive serum samples. Longitudinal changes in IgA, IgM, IgG, and Nabs. Nabs: neutralizing antibodies. **P < 0.01; ***P < 0.001

There were 219 participants who had three consecutive serum samples with positive total antibodies at baseline, of which 154 had no underlying disease and 65 had underlying disease. We compared the changes in their IgA, IgM, IgG, and neutralizing antibodies titers over time. The results showed that the IgG titers decreased significantly with or without underlying diseases and the IgA titers of people without underlying diseases decreased significantly, while the neutralizing antibody titer remained stable in people both with or without underlying disease (Fig. 1).

**Fig. 2:**
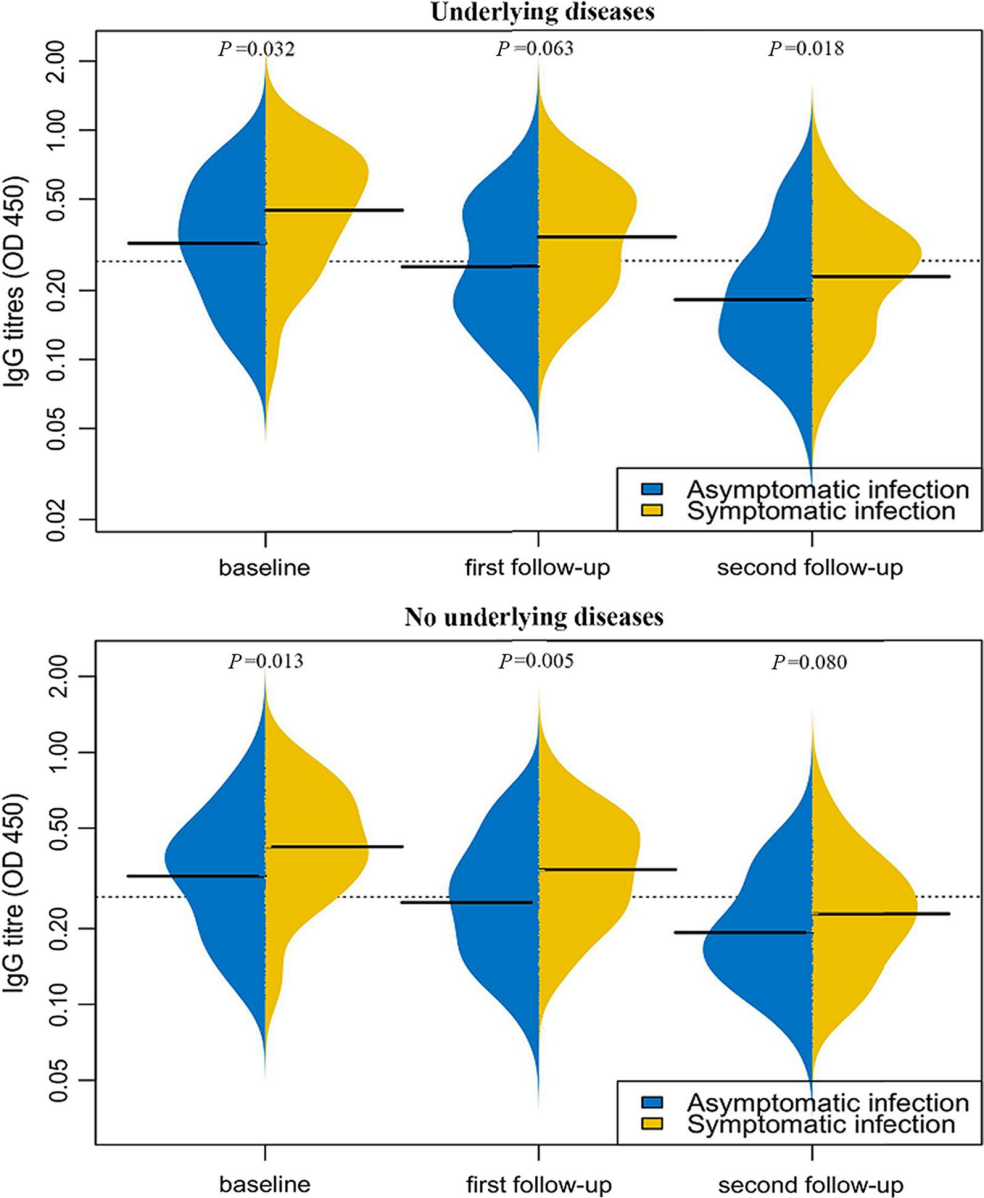
Comparison of antibody titers of symptomatic patients and asymptomatic patients among 219 subjects with positive total antibodies at baseline and three consecutive serum samples. Longitudinal changes in IgG of underlying disease group and no underlying disease group

We conducted a study on symptomatic and asymptomatic individuals, and the results showed that the proportion of IgA, IgM and IgG positive decreased gradually with the passage of time, and the proportion of neutralizing antibody positive remained stable, regardless of whether there were underlying diseases or not (Table 3). We further compared the IgG titers of symptomatic and asymptomatic patients during baseline, the first follow-up, and the second follow-up. The results showed that in the underlying diseases group, the titer of symptomatic and asymptomatic patients at baseline and the second follow-up was different, and the titer of asymptomatic patients was lower than that of symptomatic patients (baseline, *P* = 0.032, second follow-up, *P* = 0.018). In the no underlying diseases group, the titers of symptomatic and asymptomatic patients at baseline and the first follow-up were different, and the titers of asymptomatic patients were lower than those of symptomatic patients (baseline, *P* = 0.013, one follow-up, *P* = 0.005) (Fig. 2, Additional file 1: Fig. S1).

**Table 3:** When the total antibody is positive, the change in the number of symptomatic and asymptomatic positive patients with and without underlying disease

Total antibody positive (No.)	Underlying disease (No.)	IgA + (%)	IgM + (%)	IgG + (%)	Nabs + (%)
Baseline					
Symptomatic (65)	Yes (23)	5 (21.74%)	0 (0.00%)	23 (100.00%)	14 (60.87%)
	No (42)	7 (16.67%)	3 (7.14%)	42 (100.00%)	24 (57.14%)
Asymptomatic (248)	Yes (74)	17 (22.97%)	7 (9.46%)	74 (100.00%)	29 (39.19%)
	No (174)	36 (20.69%)	23 (13.22%)	174 (100.00%)	68 (39.08%)
First follow-up					
Symptomatic (56)	Yes (20)	3 (15.00%)	0 (0.00%)	20 (100.00%)	15 (75.00%)
	No (36)	3 (8.33%)	1 (2.78%)	36 (100.00%)	22 (61.11%)
Asymptomatic (156)	Yes (46)	8 (17.39%)	1 (2.17%)	46 (100.00%)	20 (43.48%)
	No (110)	13 (11.82%)	3 (2.73%)	109 (99.09%)	45 (40.91%)
Second follow-up					
Symptomatic (60)	Yes (20)	0 (0.00%)	0 (0.00%)	19 (95.00%)	13 (65.00%)
	No (40)	0 (0.00%)	0 (0.00%)	39 (97.50%)	27 (67.50%)
Asymptomatic (178)	Yes (48)	6 (12.50%)	1 (2.08%)	44 (91.67%)	23 (47.92%)
	No (130)	11 (8.46%)	3 (2.31%)	129(99.23%)	61(46.92%)

*Nabs* neutralising antibody titres

**Fig. 3:**
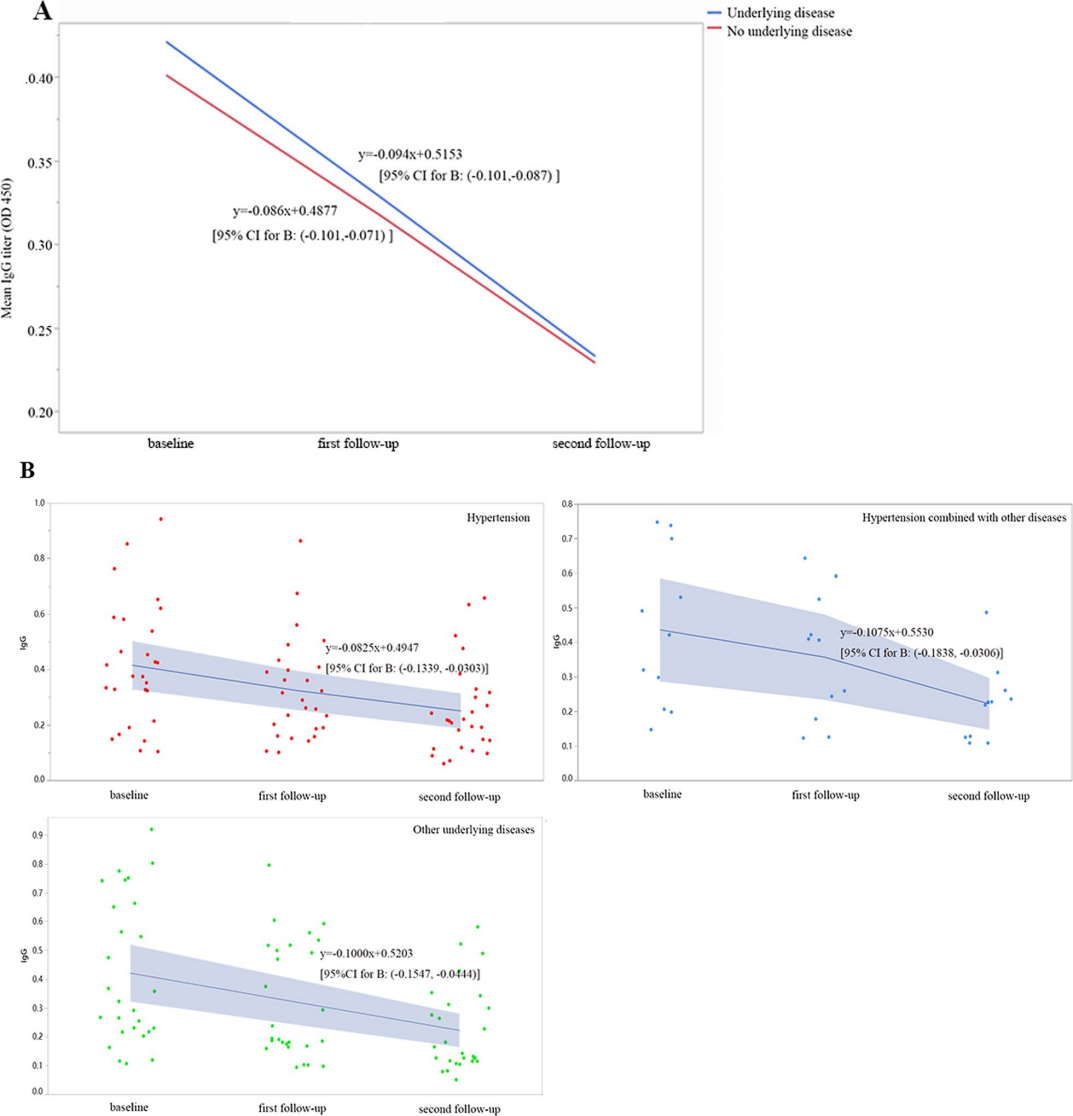
Comparison of the decline rate in IgG antibody titer among the 219 subjects with positive total antibodies at baseline and three consecutive serum samples. **A** Comparison of the decline rate between underlying diseases and no underlying diseases. **B** A total of 65 people in the underlying disease group were positive for antibodies at baseline and in three consecutive samples. Comparison of the following three groups: hypertension group, hypertension combined with other diseases group, and other underlying diseases group. **P < 0.01; ***P < 0.001; ns: no significance

Due to the obvious downward trend of IgG, we compared the decline rate of IgG titer between people with and without underlying disease. The results showed that there was no significant difference in the decline rate of IgG titer between people with underlying diseases and those without underlying diseases (Fig. 3A). A total of 65 patients with underlying diseases who had positive total antibodies at baseline and in three consecutive serum samples were divided into three groups according to the underlying disease type: simple hypertension group, hypertension combined with other diseases, and other underlying diseases group. We compared the decline rates of the IgG titers among these three groups and their results indicated no significant difference (Fig. 3B).

## Discussion

The results of this study indicate that in the case of natural infection, the seroprevalence of anti-SARS-CoV-2 antibodies in people ≥ 40 years of age with underlying diseases in Wuhan was 6.30% (95% CI [5.09–7.52]), while it was 6.12% in people without underlying diseases (95% CI [5.33–6.91]). These results suggest that the common underlying diseases as previously mentioned had no significant effect on the positive antibody conversion rate (P > 0.05). There were 219 participants who had three consecutive serum samples that were positive. Regardless of whether underlying disease was present, the IgG titer decreased significantly over time, and the neutralizing antibody titer remained relatively stable within the 9 months at least. There was no significant difference in the decline rate of IgG between people with and without underlying diseases. In patients with underlying diseases, the mean IgG titers of asymptomatic infections at the second follow-up were still lower than those of symptomatic infections, suggesting that the presence or absence of symptoms after infection is related to the intensity of immune efficacy.

There have been a few studies have proven that underlying disease is a risk factor for severe disease, but it is unknown whether they are risk factors for SARS-CoV-2 infection. Our study is the first serological study of dynamic follow-up of people 40 years of age and older with underlying diseases and in a state of natural infection. We continued to follow up on the rate of positive antibodies and their dynamic changes within the 9 months at least. At the same time, we also conducted tests on participants’ antiviral immunoglobulin and neutralizing antibodies, comprehensively assessed the immune response and efficacy, and improved understanding of the seroconversion of people with underlying diseases after natural infection with SARS-CoV-2. To the best of our knowledge, there are few long-term studies with such a large sample of people with natural infections and all-round testing of participants’ antiviral immunoglobulins and neutralizing antibodies simultaneously. With these methods, it is possible to evaluate the characteristics of the immune response to SARS-CoV-2 infection in the common underlying disease population more objectively.

We also used logistic regression (stepwise regression) regulations to avoid the collinearity problem between various factors. After adjustment, the risk of SARS-CoV-2 infection in retirees was 2.71 times that of other occupations in the group with underlying diseases, which may be related to the fact that most retirees are elderly. Wei-Jie Guan et al. conducted a study of 1,590 confirmed COVID-19 patients with an average age of nearly 50 years in 575 hospitals in 31 provinces, autonomous regions, and municipalities in mainland China [8]. The results showed that COVID-19 patients with at least one comorbidity were associated with greater severity of the disease [8]. Complications mainly included hypertension, diabetes, cerebrovascular disease, and chronic obstructive pulmonary disease. These results align with our research, suggesting that retirees with underlying disease were at higher risk of infection. As a special population, health workers have higher exposure risks and were more likely to be infected with SARS-CoV-2 (OR = 17.76, 95%, CI: 3.32–94.95). The confidence interval range was larger because the number of qualified medical personnel included in the statistical analysis was small. However, it still provided a certain amount of practical reference value. In a cross-sectional study from the Lombardy region in Italy, IgG serological testing was conducted on health workers from April 1 to May 26, 2020 [9]. The results showed that some professionals accustomed to managing infectious diseases had a higher risk of SARS-CoV-2 infection [9]. This is related to the repeated exposure of health workers to COVID-19 patients. One should be reminded that attention must be paid to the personal protection of key populations to reduce the risk of infection in the occupational environment, including current immigration management and related staff. People who have been exposed to fever or respiratory symptoms since December 2019 are more susceptible to SARS-CoV-2 infection because these people were more likely to be exposed to SARS-CoV-2.

Similar to SARS-CoV and the Middle East Respiratory Syndrome Coronavirus (MERS-COV), SARS-CoV-2 infection can stimulate the humoral immune response [10–12]. Understanding the positive rate of antibodies on a population-level and the dynamic characteristics of humoral immunity is critical to formulating vaccination strategies, especially for middle-aged and elderly people with common underlying diseases. The key to controlling COVID-19 is the development of effective vaccines [13]. Although a variety of effective vaccines are currently available in China, such as inactivated vaccines, adenovirus vector vaccines, and subunit recombinant protein vaccines, the task of vaccination is still arduous in a large number of middle-aged and elderly people with underlying diseases, for which the scientific formulation of vaccination strategies and procedures is especially important. Therefore, it is essential to further detect IgG antibodies and neutralizing antibodies of SARS-CoV-2 to assess the dynamic changes in the attenuation of the immune function in patients with underlying disease over a long period of time.

In our study, the positive conversion rate of neutralizing antibodies of people with underlying diseases was higher than that of those without underlying diseases at each stage of the test. In people with and without underlying diseases, we have observed that the positive conversion rate of neutralizing antibodies in symptomatic patients at all stages was higher than that of asymptomatic patients. This result may be related to patients with underlying diseases being more likely to be infected with SARS-CoV-2 and become symptomatic infections [14, 15].

The neutralizing antibody response helps to prevent reinfection with the virus [16, 17]. A study in Iceland showed that the total antibody titer did not decrease significantly within 4 months after the infection was confirmed [18]. A study by Cesheng Li et al. included 869 patients who recovered from natural infections in Wuhan, from whom 1782 plasma samples were collected and analyzed [16]. The results showed that more than 70% of plasma donors could continue to produce detectable receptor-binding domain (RBD)-IgG for more than 1 year after diagnosis [19]. Our research found that the IgG titer decreased significantly regardless of the underlying disease, while the neutralizing antibody titer remained stable within the 9 months at least. Regardless of whether the antibody-positive patients had symptoms, the neutralizing antibody titer did not decrease significantly during the study period in either groups (underlying disease and non-underlying disease), suggesting that it may be unnecessary to differentiate between these two groups of people in the future vaccination process.

Some studies have also performed a linear fit of antibody titers. However, there is currently no study comparing the decline rate of IgG titers in positive patients with different underlying diseases. Therefore, we compared the decline rate of IgG in people with and without underlying diseases. The IgG titer of people with underlying disease declined slightly, but there was no difference between the regression coefficients of the two groups. Therefore, the immune protection after receiving vaccination may not have a significant impact, and there is no need to formulate a special immunization program for people with underlying diseases.

We also studied symptomatic and asymptomatic patients. Regardless of whether the two groups had underlying diseases, the proportion of people positive for IgA, IgM, and IgG gradually decreased over time, while the ratio of neutralizing antibodies was relatively stable. Combined with the results of our previous studies, it indicates that asymptomatic infections in the real world will likely be an important part of the immune population. Therefore, a scientific and objective understanding of their antibody levels and changes is critical for formulating future vaccination strategies and procedures for such populations. It is also the key to preventing the continuous spread of the COVID-19 pandemic and the continuous mutation of the virus as soon as possible.

The constant mutation of the virus has caused concern worldwide because it may spread more quickly, and the effectiveness of vaccines against these mutated viruses may also be reduced, especially for middle-aged and elderly people with underlying diseases [20]. A Danish study showed that previous infections could only provide 47% protection in people 65 years of age and older, indicating that the elderly are more likely to be infected with COVID-19 again [21]. An Israeli study showed that, including the elderly, two doses of Pfizer’s BioNTech COVID-19 vaccine has a 95% protection rate against infection, hospitalization, severe illness, and death [22]. However, as the main population of basic diseases are elderly, studies on whether different vaccination strategies should be formulated to improve the effectiveness of protection have been lacking. Our research suggests that as long as the vaccination conditions are met, the presence or absence of underlying diseases will not significantly affect post-vaccination immunity and effectiveness. In any case, taking personal protective measures, maintaining a safe social distance, and vaccinating as soon as possible are still the most reliable prevention and control measures in the context of the continuing COVID-19 pandemic.

This study also has some limitations. First, some symptomatic patients reported that they had symptoms, in which case recall bias may have occurred. Second, after the Wuhan unlockdown, we followed up with the patients, but there were quite a few asymptomatic people with infection, and we could not determine the time of their initial infection. Third, since more than half of the infected people in mainland China are concentrated in Wuhan at that time, the extrapolation of the results may be limited, although this limitation is likely small. Fourth, we found that after two follow-up visits, there were 65 SARS-CoV-2 patients who had underlying diseases and three consecutive positive serum samples. Since the underlying diseases of these 65 COVID-19 patients were relatively scattered, we were unable to stratify the comorbidities and observe the immune response.

Under the current continuous pandemic situation, the elderly in China have started to receive vaccinations with full coverage. This group may be the biggest beneficiary of vaccination. In the future, it is still necessary to conduct more targeted scientific research on the humoral immune durability of anti-SARS-CoV-2 antibodies of the elderly under different vaccination conditions.

## Conclusion

In sum, our work focused on the serological study of people with underlying diseases and in a state of natural infection of original strain. Regardless of the underlying diseases, the IgG titer decreased significantly over time, while there was no significant difference in the decline rate of IgG between the two groups; and the neutralizing antibody titer remained relatively stable within the 9 months at least. Regardless of the presence or absence of underlying diseases, the mean IgG titer was lower in asymptomatic patients with infection than in symptomatic patients.

## Supplementary Information

**Additional file 1: Figure S1.** Changes of IgG titers in symptomatic and asymptomatic infections over time. A Underlying diseases, IgG titer changes in patients with symptomatic infection. B Underlying diseases, IgG titer changes in patients with asymptomatic infection. C No underlying diseases, IgG titer changes in patients with symptomatic infection. D No underlying diseases, IgG titer changes in patients with asymptomatic infection.

**Additional file 2: Table S1.** Multivariate logistic regression to explore the risk factors that affect people to contract the SARS-CoV-2. **Table S2.** Multivariate logistic regression to explore the risk factors that affect people with underlying diseases to contract the SARS-CoV-2. **Table S3.** Demographics and positive rate of total antibodies of study subjects.

## Data Availability

The datasets used and/or analysed during the current study are available from the corresponding author on reasonable request.

## Abbreviations

ECLIA: Electrochemiluminescence immunoassay
ELISA: Enzyme linked immunosorbent assay
OD_45_: Optical density at 450 nm
CI: Confidence interval
OR: Odds ratio
