# Correction to: Seroprevalence and dynamics of anti-SARS-CoV-2 antibodies: a longitudinal study based on patients with underlying diseases in Wuhan

**DOI:** 10.1186/s12931-022-02127-1

**Published:** 2022-08-25

**Authors:** Jin Yang, Libing Ma, Li Guo, Ting Zhang, Zhiwei Leng, Mengmeng Jia, Fangyuan Chen, Weiran Qi, Xingxing Zhang, Qing Wang, Yuan Yang, Luzhao Feng, Lili Ren, Weizhong Yang, Chen Wang

**Affiliations:** 1 School of Population Medicine and Public Health, Chinese Academy of Medical Sciences & Peking Union Medical College, Beijing, China; 2 Institute of Pharmaceutical and Medical Devices Supervision, National Medical Products Administration-Chinese Academy of Medical Sciences, Beijing, China; 3 Department of Respiratory and Critical Care Medicine, Affiliated Hospital of Guilin Medical University, Guilin, China; 4 National Health Commission Key Laboratory of Systems Biology of Pathogens and Christophe Mérieux Laboratory, Institute of Pathogen Biology, Chinese Academy of Medical Sciences & Peking Union Medical College, Beijing, China; 5 Key Laboratory of Respiratory Disease Pathogenomics, Chinese Academy of Medical Sciences and Peking Union Medical College, Beijing, China

## Correction to: Respiratory Research (2022) 23:188 10.1186/s12931-022-02096-5

Following publication of the original article [1], the authors would like to correct the article note of corresponding author information and equal contribution texts. It has been corrected in this correction.

^†^Jin Yang, ^†^Libing Ma and ^†^Li Guo contributed equally to this work.

*Lili Ren, *Weizhong Yang and *Chen Wang were the corresponding authors.

The original article [1] has been corrected.
