# Supplementary figures and images for: Seroprevalence and dynamics of anti-SARS-CoV-2 antibodies: a longitudinal study based on patients with underlying diseases in Wuhan
Source: Respir Res. 2022 Jul 15;23:188. doi: 10.1186/s12931-022-02096-5 (PMC9284953; doi:10.1186/s12931-022-02096-5)

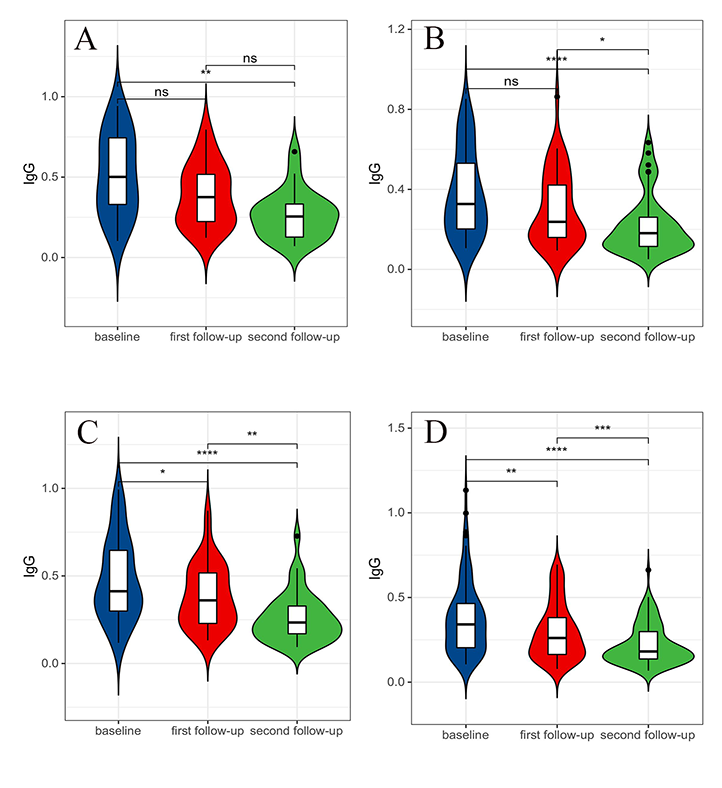

Supplement: Supplementary file 1 — Additional file 1: Figure S1. Changes of IgG titers in symptomatic and asymptomatic infections over time. A Underlying diseases, IgG titer changes in patients with symptomatic infection. B Underlying diseases, IgG titer changes in patients with asymptomatic infection. C No underlying diseases, IgG titer changes in patients with symptomatic infection. D No underlying diseases, IgG titer changes in patients with asymptomatic infection. [file 12931_2022_2096_MOESM1_ESM.tif]
