# Supplementary material for: Seroprevalence and dynamics of anti-SARS-CoV-2 antibodies: a longitudinal study based on patients with underlying diseases in Wuhan
Source: Respir Res. 2022 Jul 15;23:188. doi: 10.1186/s12931-022-02096-5 (PMC9284953; doi:10.1186/s12931-022-02096-5)
Supplement: Supplementary file 2 — Additional file 2: Table S1. Multivariate logistic regression to explore the risk factors that affect people to contract the SARS-CoV-2. Table S2. Multivariate logistic regression to explore the risk factors that affect people with underlying diseases to contract the SARS-CoV-2. Table S3. Demographics and positive rate of total antibodies of study subjects. [file 12931_2022_2096_MOESM2_ESM.docx]

**Supplementary materials**

**Tables**

**Table S1 Multivariate logistic regression to explore the risk factors that affect people to contract the SARS-CoV-2**

| **Variables** | **P-value** | **OR** | **95%CI** |
| --- | --- | --- | --- |
| **Age (years)** |  |  |  |
| 40-45 | 1.000 | | |
| 46-50 | 0.147 | 1.277 | 0.918-1.778 |
| 51-55 | 0.645 | 1.092 | 0.752-1.586 |
| 56-60 | 0.155 | 1.315 | 0.901-1.919 |
| 61-65 | 0.911 | 0.976 | 0.637-1.495 |
| ≥66 | 0.635 | 1.108 | 0.726-1.692 |
| **Gender** |  |  |  |
| Male | 1.000 | | |
| Female | 0.459 | 1.100 | 0.855-1.415 |
| **Occupation** |  | | |
| Others | 1.000 | | |
| Retirees | 0.466 | 1.132 | 0.811-1.580 |
| Workmen/farmers | **0.021** | 0.692 | 0.506-0.947 |
| Commercial service personal | 0.325 | 0.825 | 0.563-1.209 |
| Community workers | **0.003** | 0.502 | 0.319-0.792 |
| Health workers | 0.886 | 1.076 | 0.396-2.922 |
| **Smoke** |  | | |
| No | 1.000 | | |
| Yes, haven't quit smoking | 0.080 | 0.770 | 0.575-1.032 |
| Yes, have quit smoking | 0.791 | 1.070 | 0.648-1.767 |
| **Self reported SARS-CoV-2 symptoms** |  |  |  |
| No | 1.000 | | |
| Yes | **<0.001** | 4.803 | 3.412-6.761 |
| **Contact with anyone with fever or respiratory symptoms since December, 2019** |  |  |  |
| No | 1.000 | | |
| Yes | **<0.001** | 2.671 | 1.852-3.851 |
| **Underlying disease** |  |  |  |
| No | 1.000 | | |
| Yes | 0.360 | 0.895 | 0.706-1.135 |

**Table S2 Multivariate logistic regression to explore the risk factors that affect people with underlying diseases to contract the SARS-CoV-2**

| **Variables** | **Underlying diseases** | | | |
| --- | --- | --- | --- | --- |
|  | ***P*-value** | **OR** | **95%CI** | |
| **Occupation** |  |  |  |  |
| Others | 1.000 | | | |
| Retirees | **0.003** | 2.710 | 1.397-5.256 | |
| Workmen/farmers | 0.847 | 1.085 | 0.475-2.481 | |
| Commercial service personal | 0.604 | 0.709 | 0.193-2.606 | |
| Community workers | 0.314 | 0.447 | 0.093-2.141 | |
| Health workers | **0.001** | 17.764 | 3.323-94.951 | |
| **Contact with anyone with fever or respiratory symptoms since December, 2019** |  |  |  |  |
| No | 1.000 | | | |
| Yes | **<0.001** | 5.777 | 2.985-11.183 | |

OR, odd ratio; CI, confidence interval.

**Table S3. Demographics and positive rate of total antibodies of study subjects**

| **Variables** | **Number of population (%) at baseline** | **Number of positive total antibody at baseline (%)** | **Number of population at first follow-up(%)** | **Number of positive total antibody at first follow-up (%)** | **Number of population at second follow-up(%)** | **Number of positive total antibody at second follow-up (%)** |
| --- | --- | --- | --- | --- | --- | --- |
| Total | 5067 | 313 (6.18%) | 1396 | 212(15.19%) | 1402 | 238(16.98%) |
| **Age (years)** |  |  |  |  |  |  |
| 40-45 | 1072 (21.16%) | 57 (5.32%) | 275(19.70%) | 38 (13.82%) | 274(19.54%) | 45 (16.42%) |
| 46-50 | 924 (18.24%) | 48 (5.19%) | 221(15.83%) | 36 (16.29%) | 221(15.76%) | 44 (19.91%) |
| 51-55 | 737 (14.55%) | 45 (6.11%) | 196(14.04%) | 28 (14.29%) | 187(13.34%) | 33 (17.65%) |
| 56-60 | 767 (15.14%) | 63 (8.21%) | 227(16.26%) | 41 (18.06%) | 228(16.26%) | 45 (19.74%) |
| 61-65 | 740 (14.60%) | 37 (5.00%) | 219(15.69%) | 27 (12.33%) | 228(16.26%) | 30 (13.16%) |
| ≥66 | 827 (16.32%) | 63 (7.62%) | 258(18.48%) | 42 (16.28%) | 264(18.83%) | 41 (15.53%) |
| **Sex** |  |  |  |  |  |  |
| Male | 2492 (49.18%) | 124 (4.98%) | 633(45.34%) | 81 (12.80%) | 647(46.15%) | 94 (14.53%) |
| Female | 2575 (50.82%) | 189 (7.34%) | 763(54.66%) | 131 (17.17%) | 755(53.85%) | 144 (19.07%) |
| **Occupation** |  |  |  |  |  |  |
| Commercial service personal | 546 (10.78%) | 28 (5.13%) | 133(9.53%) | 21 (15.79%) | 132 (9.42%) | 25 (18.94%) |
| Workmen/farmers | 1270 (25.06%) | 40 (3.15%) | 320(22.92%) | 20 (6.25%) | 325 (23.18%) | 25 (7.69%) |
| Others | 1312 (25.89%) | 87 (6.63%) | 379(27.15%) | 60 (15.83%) | 374 (26.68%) | 73 (19.52%) |
| Community workers | 425 (8.39%) | 17 (4.00%) | 72 (5.16%) | 10 (13.89%) | 78 (5.56%) | 15 (19.23%) |
| Retirees | 1476 (29.13%) | 138 (9.35%) | 477 (34.17%) | 99 (20.75%) | 477 (34.02%) | 98 (20.55%) |
| Health workers | 38 (0.75%) | 3 (7.89%) | 15 (1.07%) | 2 (13.33%) | 16 (1.14%) | 2 (12.50%) |
| **Smoke** |  |  |  |  |  |  |
| No | 3392 (66.94%) | 240 (7.08%) | 992 (71.06%) | 168 (16.94%) | 983 (70.11%) | 189 (19.23%) |
| Yes | 1446 (28.54%) | 53 (3.67%) | 347 (24.86%) | 31 (8.93%) | 361 (25.75%) | 35 (9.70%) |
| Ever smoked | 229 (4.52%) | 20 (8.73%) | 57 (4.08%) | 13 (22.81%) | 58 (4.14%) | 14 (24.14%) |
| **Self-reported symptom** |  |  |  |  |  |  |
| No | 4875 (96.21%) | 248 (5.09%) | 1308 (93.70%) | 156 (11.93%) | 1312 (93.58%) | 178 (13.57%) |
| Yes | 192 (3.79%) | 65 (33.85%) | 88 (6.30%) | 56 (63.64%) | 90 (6.42%) | 60 (66.67%) |
| **To the hospital for fever or respiratory symptoms since December 2019** |  |  |  |  |  |  |
| No | 4946 (97.61%) | 258 (5.22%) | 1333 (95.49%) | 156 (11.70%) | 1336 (95.29%) | 178 (13.32%) |
| Yes | 121 (2.39%) | 55 (45.45%) | 63 (4.51%) | 56 (88.89%) | 66 (4.71%) | 60 (90.91%) |
| **Contact with anyone with fever or respiratory symptoms since December 2019** |  |  |  |  |  |  |
| No | 4821 (95.15%) | 262 (5.43%) | 1297 (92.91%) | 168 (12.95%) | 1300 (92.72%) | 193 (14.85%) |
| Yes | 246 (4.85%) | 51 (20.73%) | 99 (7.09%) | 44 (44.44%) | 102 (7.28%) | 45 (44.12%) |
| **Contact with a SARS-CoV-2 confirmed case since December 2019** |  |  |  |  |  |  |
| No | 4922 (97.14%) | 280 (5.69%) | 1334 (95.56%) | 183 (13.72%) | 1336 (95.29%) | 208 (15.57%) |
| Yes | 145 (2.86%) | 33 (22.76%) | 62 (4.44%) | 29 (46.77%) | 66 (4.71%) | 30 (45.45%) |
| **Underlying disease** |  |  |  |  |  |  |
| No | 3528 (69.63%) | 216 (6.12%) | 936 (67.05%) | 146 (15.60%) | 954 (68.05%) | 170 (17.82%) |
| Yes | 1539 (30.37%) | 97 (6.30%) | 460 (32.95%) | 66 (14.35%) | 448(31.95%) | 68 (15.18%) |

Baseline time, from April 9–13, 2020; the first follow-up time, from June 11–13, 2020; the second follow-up time from October 9–December 5, 2020.

**Supplementary Figures**

**Figure S1 Changes of IgG titers in symptomatic and asymptomatic infections over time**

A：Underlying diseases, IgG titer changes in patients with symptomatic infection

B：Underlying diseases, IgG titer changes in patients with asymptomatic infection

C：No underlying diseases, IgG titer changes in patients with symptomatic infection

D：No underlying diseases, IgG titer changes in patients with asymptomatic infection

**Questionnaire of the seroepidemiological study in Wuhan, China**

Area: ______ District ______ Township (street)

Location of investigation: ______ community

Community number: □□

Current address: ______ Village (building) ______ Group (unit) No. ______.

Family number: □□ Personal number: □□

Survey object ID (automatically generated by the administrative system): ______

(township national standard code - community code - family code - personal code)

1. Name: ______ 2. Gender: □Male □Female

3. Mobile number: ______

4. Date of birth: ______/______/______ (DD/MM/YY)

5. ID number: ______

6. Occupation: □Scattered children □Nursery children □Student □Farmer □Workers □Administration officer □Healthcare worker □ Public health staff □Retirees □Commercial service personnel □Deliveryman □Takeout deliveryman □Security

□Police □Taxi driver □Cleaning staff □Community workers □Other

7. Categories of community COVID-19 prevention and control personnel: (only for high risk population survey areas)

□Community worker □Security □Police □Administration officer □Taxi driver

□Volunteer □Cleaning staff □ Community healthcare worker □Other______

8. Pregnant or not: □No □Yes, weeks of pregnancy: ______ Week ______Day

9. Did you give birth to a child in the past 6 months? □No □Yes, the delivery date: _______/_______/_______ (DD/MM/YY).

10. Underlying conditions (multi-choice): □None □Pulmonary diseases (such as asthma, COPD, pulmonary heart disease, pulmonary fibrosis, etc.) □Cancer chemotherapy □Hypertension □Diabetes □Cardiovascular and cerebrovascular diseases □Chronic kidney disease □Chronic liver disease □Immunodeficiency diseases □Unknow □Other______.

11. Do you smoke? □Yes, I haven't quit smoking; □Yes, I've quit smoking; □No.

12. Have you had the following clinical symptoms since December 2019?

Fever □No □Yes, the highest temperature______℃, date of onset was ______/______/______ (DD/MM/YY).

Cough □No □Yes, date of onset was ______/______/______ (DD/MM/YY).

Anhelation □No □Yes, date of onset was ______/______/______ (DD/MM/YY).

Rhinobyon □No □Yes, date of onset was ______/______/______ (DD/MM/YY).

Rhinorrhea □No □Yes, date of onset was ______/______/______ (DD/MM/YY).

Sore throat □No □Yes, date of onset was ______/______/______ (DD/MM/YY).

Shortness of breath □No □Yes, date of onset was ______/______/______ (DD/MM/YY).

Dyspnea □No □Yes, date of onset was ______/______/______ (DD/MM/YY).

Myalgia □No □Yes, date of onset was ______/______/______ (DD/MM/YY).

Pneumonia □No □Yes (lung CT or X-ray changes), date of onset was ______/______/______ (DD/MM/YY).

Other symptoms □No □Yes, please specify___________, date of onset was ______/______/______ (DD/MM/YY).

13. Have you visited a medical institution because of fever or respiratory diseases since December 2019? □Yes □No.

13.1. If the answer is “Yes”, the date was: □2019/12 □2020/1 □2020/2 □2020/3 □2020/4.

14. Have you been diagnosed with COVID-19 since December 2019? □Yes □No.

14. 1. If diagnosed, date of onset was ______/______/______ (DD/MM/YY).

(This information could be checked from the Hospital Information System).

15. Have you had the following travel or residence history since December 2019?

History of residence in Wuhan, □No □Yes, from______/______/______ (DD/MM/YY) to ______/______/______ (DD/MM/YY).

History of travel to Wuhan, □No □Yes, from______/______/______ (DD/MM/YY)

to______/______/______ (DD/MM/YY).

History of residence in Hubei province outside Wuhan, □No □Yes, from______/______/______ (DD/MM/YY) to______/______/______ (DD/MM/YY).

History of travel to Hubei province outside Wuhan, □No □Yes, from______/______/______ (DD/MM/YY) to ______/______/______ (DD/MM/YY).

History of living abroad, □No □Yes, name of the country ______,from ______/______/______ (DD/MM/YY) to ______/______/______ (DD/MM/YY).

History of travelling abroad, □No □Yes, name of the country_____, from ______/______/______ (DD/MM/YY) to ______/______/______ (DD/MM/YY).

16. Have you been in contact with anyone with fever or respiratory symptoms since December 2019?

□Yes □No.

16.1 If the answer is “Yes”, date of the last contact was ______/______/______ (DD/MM/YY).

17. Have you ever been exposed to COVID-19 confirmed cases since December 2019?

□ Yes □ No

17.1 If the answer is “Yes”, date of the last contact was ______/______/______ (DD/MM/YY). (This information could be checked from the local close contacts registration system)

18. Have you ever been exposed to asymptomatic SARS-CoV-2 infections since December 2019?

□ Yes □ No

18.1 If the answer is “Yes”, the date of last contact was ______/______/______ (DD/MM/YY). (This information could be checked from the local close contacts registration system)

Name of the investigator: ______ Mobile number: ______

Date of the investigation: ______/______/______/ (DD/MM/YY)

19. Is the respondent a COVID-19 confirmed case after checking from the Chinese Notifiable Infectious Diseases Information System?

□ Yes, the clinical severity is: □ asymptomatic □ mild □ moderate □ severe □ critical

□ no

Name of the inspector: ______ Mobile number: ______

Date of the inspection: ______/______/______/ (DD/MM/YY)

**Notes:**

In addition to the personal information part, sampling information and test results should also be recorded into the "Serological Survey" information system. Please keep a good record of sampling information and test results.

1. The report information of antibody detection of blood samples mainly includes: antibody testing method; manufacturer, name, batch number, the expiration date of the reagent; qualitative and quantitative testing results, date of testing, person performed testing, person reviewed testing results, etc.

2. Among the areas in which respiratory tract swab specimen were collected, nucleic acid testing information should be recorded into the system, mainly including testing method, manufacturer, name, batch number, the expiration date of the reagent; manufacturer of nucleic acid extraction reagent, fluorescence quantitative test, PCR instrument, qualitative and quantitative testing results, date of testing, person performed testing, person reviewed testing results, etc.
